# Immune inflammation and metabolic interactions in the pathogenesis of diabetic nephropathy

**DOI:** 10.3389/fendo.2025.1602594

**Published:** 2025-07-08

**Authors:** Guangjian Hou, Youzi Dong, Yuehua Jiang, Wenbo Zhao, Le Zhou, Shengnan Cao, Wei Li

**Affiliations:** ^1^ Shandong University of Traditional Chinese Medicine, Jinan, Shandong, China; ^2^ The Affiliated Hospital of Shandong University of Traditional Chinese Medicine, Jinan, Shandong, China; ^3^ Shandong First Medical University (Shandong Academy of Medical Sciences), Jinan, Shandong, China

**Keywords:** diabetic nephropathy, immune-inflammatory response, metabolic signaling pathways, kidney damage, immune cells

## Abstract

Diabetic nephropathy (DN) is a significant microvascular complication of diabetes, substantially contributing to the global prevalence of end-stage renal disease. The pathogenesis of DN is multifactorial, involving both immune-inflammatory responses and metabolic dysregulation. Hyperglycemia, a hallmark of diabetes, initiates kidney damage through various mechanisms, including oxidative stress, the accumulation of advanced glycation end products (AGEs), and changes in renal blood flow. These processes lead to the hallmark pathological features of DN, such as glomerulosclerosis and tubulointerstitial fibrosis. The immune system, particularly macrophages, T cells, and B cells, plays a crucial role in the progression of kidney injury, with inflammatory cytokines such as TNF-α and IL-6 promoting renal inflammation and fibrosis. In addition, metabolic disturbances, notably insulin resistance and dysfunction in insulin signaling, contribute to kidney dysfunction through several key signaling pathways, including PI3K/Akt, mTOR, Wnt/β-catenin, JAK/STAT, and NF-κB. The interplay between immune responses and metabolic signaling exacerbates kidney damage, creating a feedback loop that accelerates the progression of DN. While current therapeutic strategies mainly focus on managing blood glucose levels and inflammation, emerging treatments, such as GLP-1 receptor agonists and SGLT2 inhibitors, show promise in addressing both the metabolic and inflammatory aspects of the disease. Future research should focus on unraveling the complex interactions between immune and metabolic pathways to develop more targeted and personalized treatments for DN. This review highlights the significance of these mechanisms in the pathophysiology of DN and calls for innovative therapeutic approaches to combat this debilitating condition.

## 1 Introduction

Diabetic nephropathy (DN) is the leading cause of end-stage renal disease (ESRD) worldwide, representing a severe complication of diabetes mellitus. The global increase in diabetes cases has led to a rise in DN occurrences, causing significant renal dysfunction. This condition not only damages kidney function but also negatively impacts cardiovascular health, contributing to higher morbidity and mortality rates among affected individuals. Strict blood glucose control is crucial, as DN presents substantial medical challenges (1). Additionally, managing various metabolic parameters and blood pressure is essential to reduce the speed of disease progression and minimize associated medical complications (2).

The pathogenesis of diabetic nephropathy (DN) is influenced by a combination of metabolic, hemodynamic, and inflammatory factors. Immune cells, particularly macrophages and T-lymphocytes, play a crucial role in driving inflammation by releasing pro-inflammatory cytokines and chemokines, which significantly contribute to DN development and progression. These immune responses exacerbate renal damage as immune cells infiltrate the kidney tissue (3), leading to further injury. The heightened inflammation, mediated by these cytokines and chemokines (4), is closely linked to fibrotic changes in the kidney, worsening the disease process.

Additionally, DN is strongly influenced by disruptions in metabolic signaling pathways, particularly those regulating glucose and lipid metabolism. Hyperglycemia induces the formation of advanced glycation end-products (AGEs), which, together with oxidative stress, activate inflammatory pathways that accelerate renal injury (5). This creates a vicious cycle where immune responses and metabolic dysregulation continuously interact, further speeding up the progression of DN (6).

The development of targeted therapeutic strategies for diabetic nephropathy (DN) is grounded in understanding the connection between immune-inflammatory responses and metabolic signaling pathways (7). Ongoing research is focused on identifying new biomarkers and therapeutic targets that can intervene in these pathways to slow or prevent DN progression (8). Both preclinical and clinical studies have investigated anti-inflammatory therapies aimed at specific metabolic pathways (9), showing that such interventions can improve treatment outcomes for DN patients.

**Figure 1:**
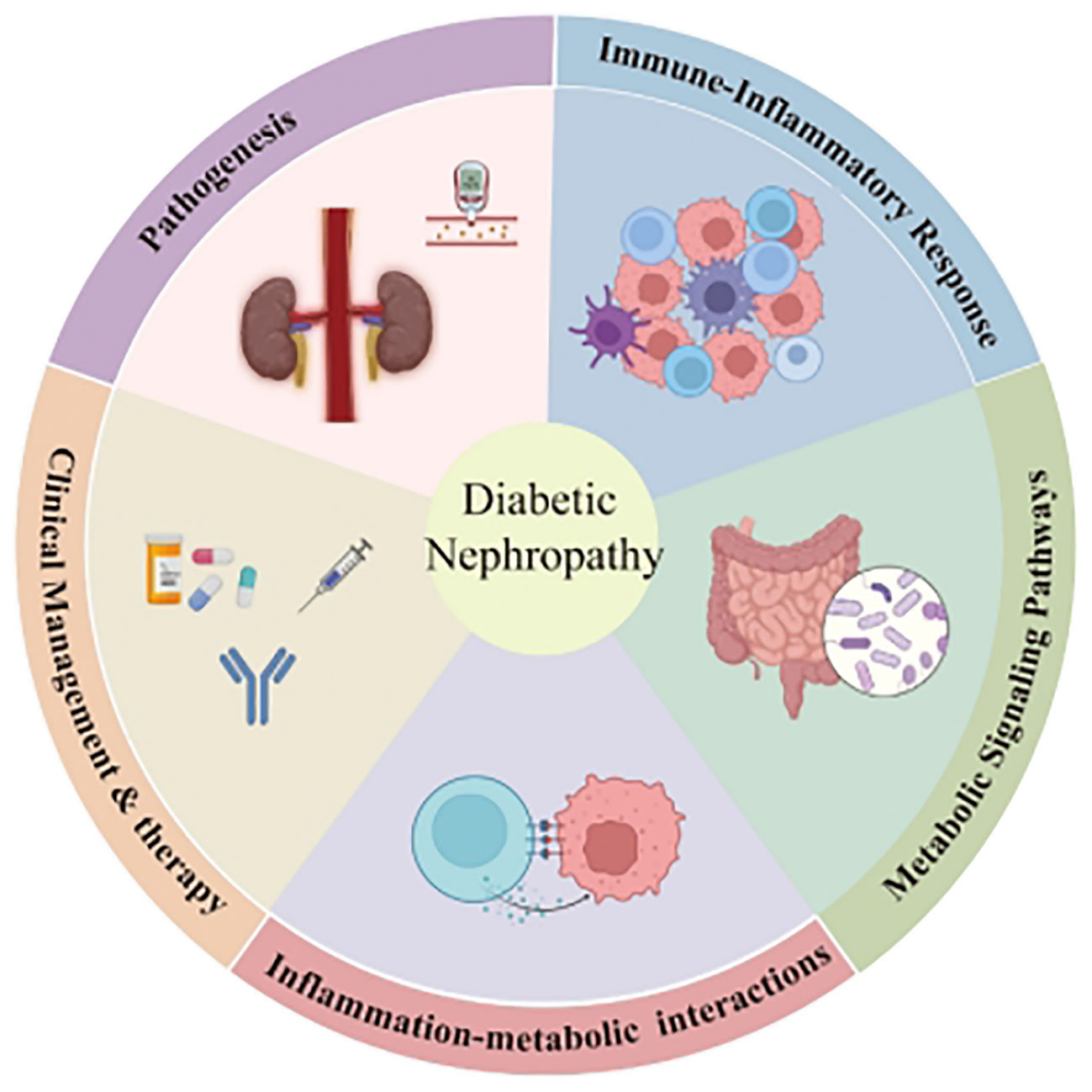
Comprehensive diagram illustrating the multifactorial pathogenesis of diabetic nephropathy (DN). Sustained hyperglycemia initiates oxidative stress and generates advanced glycation end products (AGEs), activating inflammatory pathways (e.g., NF-κB, JAK/STAT) and oxidative stress mechanisms. These processes subsequently promote immune cell infiltration and cytokine release, contributing to inflammation and fibrosis within renal tissue. The dysregulation of key metabolic signaling pathways, including PI3K/Akt, mTOR, and Wnt/β-catenin, exacerbates these effects, leading to structural damage (glomerulosclerosis, tubulointerstitial fibrosis) and progressive renal dysfunction. ROS, reactive oxygen species; NF-κB, nuclear factor kappa B; EMT, epithelial-to-mesenchymal transition.

The complex pathophysiology of DN has created significant financial and medical challenges for healthcare systems, making it a major global health issue, as illustrated in **Figure 1**. Research into the immune and metabolic pathways central to DN remains pivotal, as these efforts aim to identify mechanisms that can be targeted for therapeutic benefit (10). Continued development of treatment strategies is critical to improving the quality of life for patients suffering from advanced stages of the disease. **Figure 1** and references further support these findings.

## 2 Pathogenesis of diabetic nephropathy

The pathogenesis of diabetic nephropathy (DN) is multifactorial and complex, involving multiple interacting processes. As clearly illustrated in **Figure 1**, sustained hyperglycemia initiates several pathophysiological pathways, including oxidative stress, inflammation, metabolic dysregulation, and fibrotic changes, ultimately leading to progressive renal injury.

### 2.1 The relationship between hyperglycemia and diabetic nephropathy & mechanisms of diabetes-induced kidney injury

Hyperglycemia plays a central role in diabetic nephropathy (DN) by directly damaging renal tubules and mesangial cells through mitochondrial dysfunction and oxidative stress (11, 12). When combined with renin-dependent hypertension, high blood glucose leads to pronounced glomerulosclerosis and tubulointerstitial fibrosis, whereas diabetes alone produces only minimal histological changes (13). Moreover, persistent hyperglycemia triggers oxidative stress, inflammation, and apoptosis, along with epigenetic modifications (metabolic memory) that further accelerate DN progression (14).

Diabetic kidney disease (DKD) is multifactorial. In addition to glucose toxicity, impaired energy metabolism, chronic inflammation, and lipid metabolic disturbances contribute significantly to renal injury (15). Mitochondrial oxidative stress in glomerular endothelial cells disrupts the filtration barrier (16), while cytokine-driven inflammatory cascades exacerbate tissue damage (17). Furthermore, increased lipid toxicity and environmental factors, such as ozone exposure, disturb lipid balance and water-electrolyte homeostasis, worsening kidney injury (18, 19).

### 2.2 Classical clinical manifestations of diabetic nephropathy and its pathologic changes

Diabetic nephropathy (DN) is a severe complication of diabetes that presents distinct clinical and pathological features crucial for diagnosis. A key diagnostic marker for DN is the continuous presence of albuminuria, which, along with a reduced glomerular filtration rate (GFR), reliably indicates diabetic kidney disease. Without appropriate treatment, DN progresses to chronic kidney disease (CKD) and eventually to end-stage renal disease (ESRD), a common outcome for patients with advanced DN (20). Structurally, DN leads to several pathological changes in the kidney, including thickening of the glomerular basement membrane, expansion of the mesangial area contributing to glomerulosclerosis, and tubulointerstitial fibrosis and arteriolar hyalinosis, which further alter kidney structure. In clinical practice, the progression of DN is reflected by elevated albumin excretion rates and reduced GFR, although these measurements vary significantly due to ongoing structural changes in the kidney (21). In patients with type 2 diabetes, kidney lesions are often more varied, with some showing more severe vascular and chronic tubulointerstitial damage compared to non-diabetic glomerulopathy. Oxidative stress and the renin-angiotensin system (RAS) play pivotal roles in the pathogenesis of DN, with studies showing that activation of the oxidative stress/angiotensinogen/RAS axis accelerates nephropathy progression in diabetic kidneys (22).

Additionally, growing attention is being paid to cases of chronic kidney disease in diabetic patients who do not exhibit the typical sign of proteinuria. These patients experience a decline in kidney function without the classic signs of proteinuria, which is particularly common in older individuals and those receiving RAS inhibitors. Despite significant glomerular damage, these patients often have a slower progression to ESRD (23). Recognizing and understanding these atypical presentations is crucial for improving the diagnosis and treatment of DN.

## 3 The role of the immune inflammatory response in diabetic nephropathy

### 3.1 Immune cell-mediated inflammatory response in diabetic nephropathy

**Figure 2:**
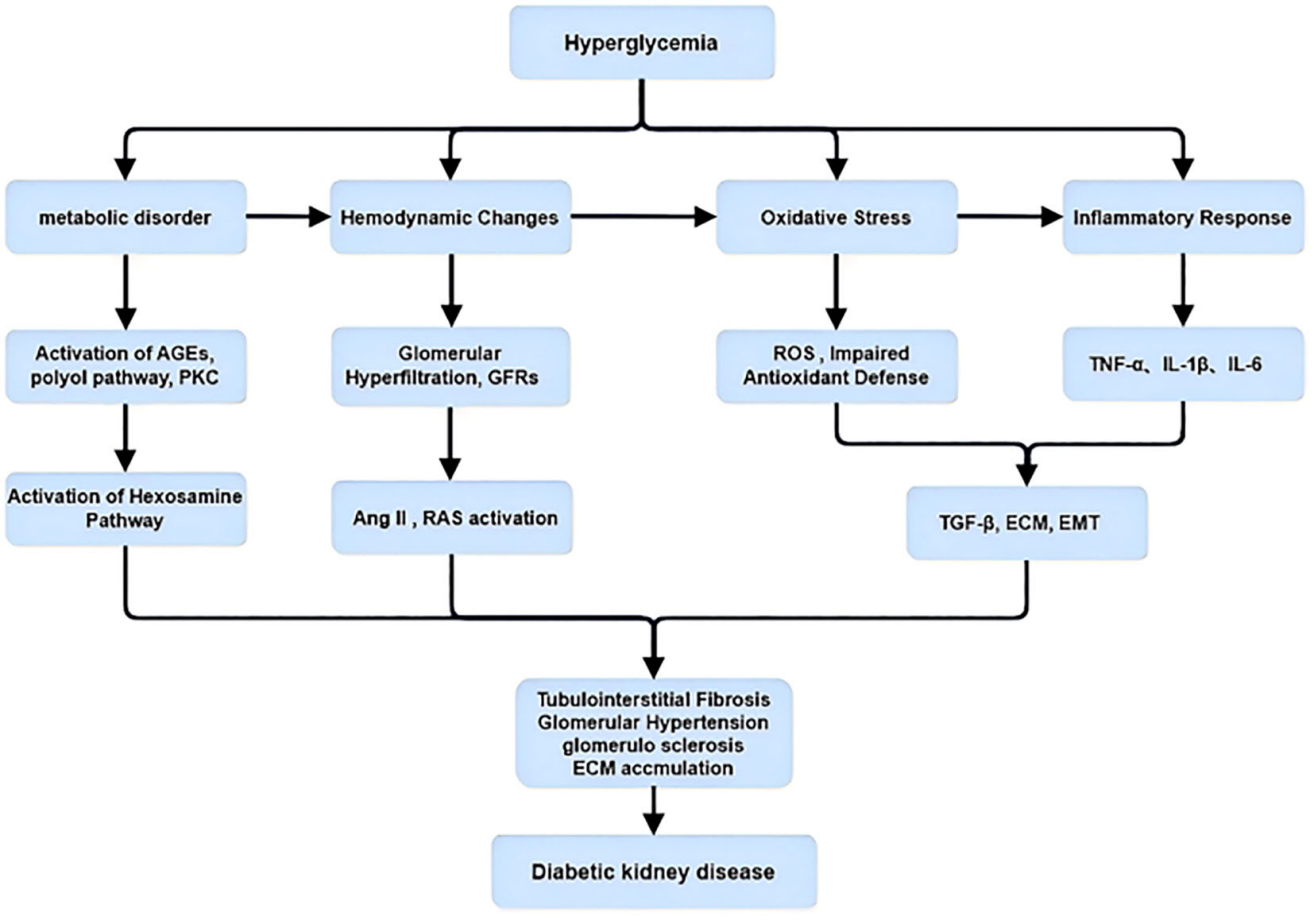
Overview of hyperglycemia-induced pathogenic pathways in diabetic kidney disease. Hyperglycemia triggers metabolic disorders, hemodynamic alterations, oxidative stress, and inflammatory responses. These processes lead to activation of signaling pathways including AGEs, PKC, RAS, ROS, and TGF-β, ultimately promoting tubulointerstitial fibrosis, glomerular hypertension, ECM accumulation, and progression to diabetic kidney disease.

Diabetic nephropathy (DN) is increasingly recognized as a chronic inflammatory condition in which immune cell infiltration contributes to glomerular and tubular injury(show in **Figure 2**). Among the immune cells involved, macrophages, T cells, and B cells have been extensively studied for their pathogenic roles.

Macrophages are the most abundant immune cells found in diabetic kidneys. Hyperglycemia and advanced glycation end products (AGEs) drive macrophage polarization toward the pro-inflammatory M1 phenotype. These M1 macrophages secrete cytokines such as TNF-α, IL-1β, and IL-6, which induce mesangial matrix expansion, promote tubular epithelial-to-mesenchymal transition, and contribute to interstitial fibrosis. Animal models have shown that inhibition of macrophage recruitment or skewing toward the anti-inflammatory M2 phenotype can significantly reduce renal injury (24, 25).

T cells also play a crucial role. Studies have identified increased infiltration of CD4^+^ T helper cells, particularly the Th1 and Th17 subsets, in diabetic kidneys. These cells secrete IFN-γ and IL-17, respectively, which perpetuate the local inflammatory environment by stimulating macrophage activation and endothelial dysfunction. In contrast, regulatory T cells (Tregs), which are typically involved in suppressing immune responses, are functionally impaired in DN, leading to immune imbalance and persistent inflammation (26, 27).

B cells contribute both through humoral immunity and antigen presentation. Aside from producing autoantibodies that may contribute to glomerular injury, B cells act as antigen-presenting cells (APCs) and secrete cytokines that modulate T cell activity. Recent evidence suggests that depletion of B cells can attenuate albuminuria and histological damage in mouse models of DN (28).

The interactions among these immune cell subsets create a feed-forward inflammatory loop that exacerbates kidney damage. Notably, many of these immune responses are metabolically regulated. For instance, pro-inflammatory immune cells in DN tend to rely on aerobic glycolysis, whereas anti-inflammatory cells such as Tregs and M2 macrophages depend on fatty acid oxidation and mitochondrial oxidative phosphorylation—a concept known as immunometabolic reprogramming (27, 29). This highlights the need for therapies that simultaneously target both inflammation and metabolic dysfunction in diabetic nephropathy.

### 3.2 The role of immunoinflammation in tubular and glomerular injury

**Figure 3:**
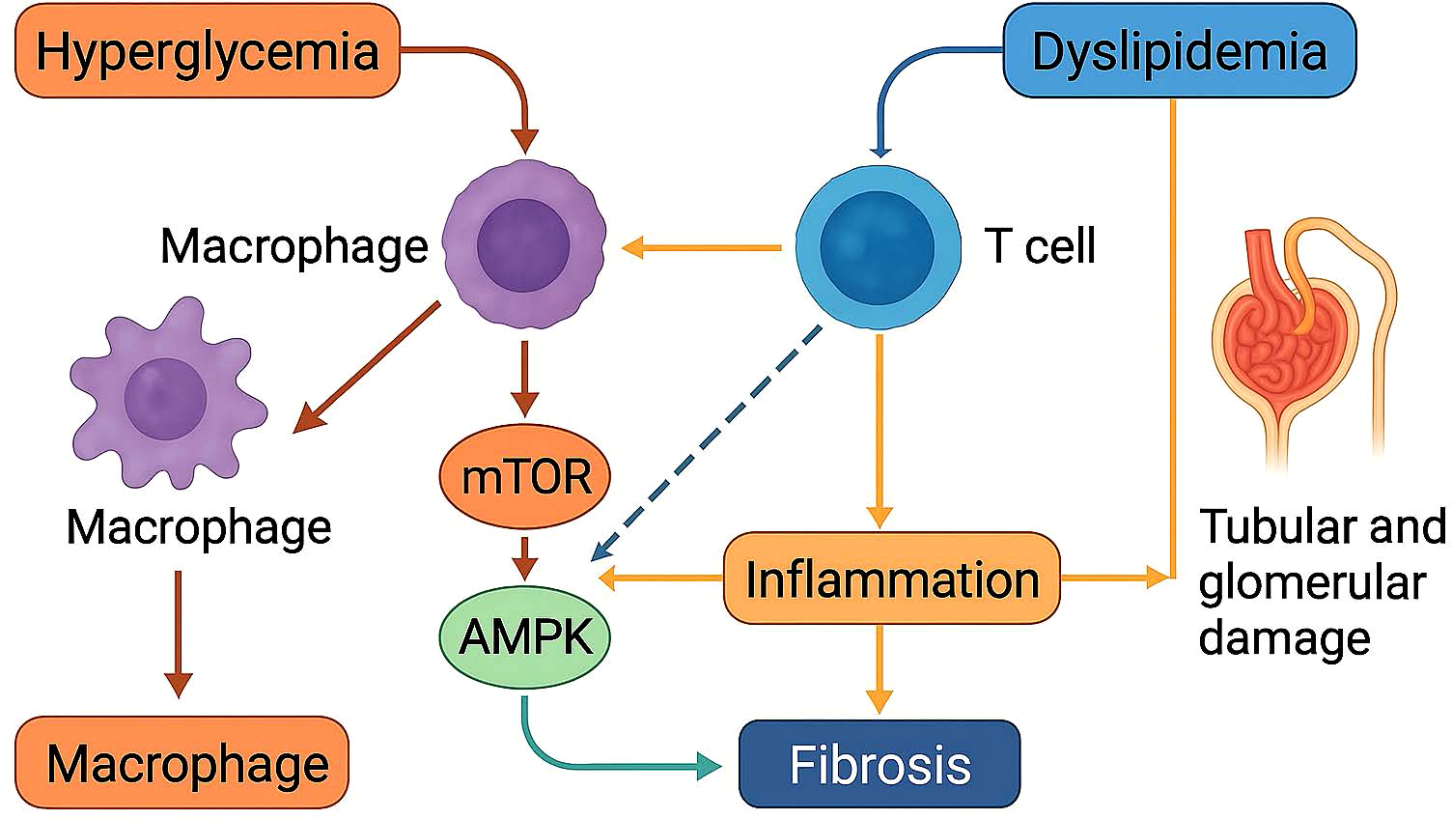
Immune-metabolic interactions contributing to tubular and glomerular injury in diabetic nephropathy. Hyperglycemia activates macrophages, which promote mTOR signaling and influence AMPK activity. Concurrently, dyslipidemia activates T cells, amplifying inflammation. The combined effects of macrophage and T cell activation lead to fibrosis and progressive renal damage. AMPK may exert a compensatory anti-inflammatory effect, modulating fibrosis development.

Inflammatory immunity plays a critical role in tubular and glomerular damage(show in **Table 1**, **Figure 3**). Tubular injury is associated with multiple factors, including hypoxia, proteinuria, toxins, metabolic disorders, and aging. While tubules were traditionally viewed as passive victims of damage, recent studies have revealed that they actively contribute to kidney disease progression. When tubular epithelial cells are damaged, they become inflammatory and fibrotic, releasing bioactive molecules that drive interstitial inflammation and fibrosis (30).

**Table 1:** Immune-inflammatory response in diabetic nephropathy.

Aspect	Key Components	Mechanisms	Impact on DN Progression
Immune Cell-Mediated Inflammation in DN	Macrophages, T cells, B cells	Macrophages release cytokines (TNF-α, IL-6) → chronic inflammation → fibrosis.	Exacerbates renal fibrosis and podocyte injury.
Role of Inflammation in Tubular and Glomerular Injury	Tubular epithelial cells, podocytes, mesangial cells	Inflammatory response → oxidative stress → tubular and glomerular cell apoptosis.	Leads to progressive loss of kidney function.
Expression and Function of Inflammatory Cytokines	TNF-α, IL-6, IL-19, MCP-1	TNF-α, IL-6 trigger NF-KB signaling → sustained inflammation → kidney injury.	Sustained inflammation accelerates nephropathy progression.
Autoimmune Responses and DN	Autoantibodies, immune complexes, adaptive immune response	Dysregulated immune tolerance → autoantibody production → kidney damage.	Potentially contributes to treatment resistance and chronic kidney disease.

In the glomeruli, immune inflammation is similarly crucial. Angiotensin II significantly contributes to glomerular inflammation in the pathogenesis of glomerular diseases. In autoimmune diseases, the activation of type 1 angiotensin receptors worsens glomerular inflammation and damage (31). T cells also play a vital role in autoimmune nephropathy, either by supporting B cell differentiation and antibody production or by directly causing inflammation and cytotoxic damage to renal cells (32).

In acute kidney injury (AKI), both lymphocytes and innate immune cells drive the complex inflammatory process within the kidneys. Studies show that CD4+ and CD8+ T cells, B cells, and neutrophils may be involved in the development and progression of AKI, while regulatory T cells, double-negative T cells, and type 2 innate lymphocytes offer protective effects (33). These immune cells participate in both the injury and repair processes during AKI and contribute to dysfunction in distant organs.

### 3.3 Expression and function of inflammatory factors in diabetic nephropathy

In diabetic nephropathy (DN), multiple inflammatory mediators orchestrate tissue injury. Among them, tumor necrosis factor-α (TNF-α), interleukin-6 (IL-6), interleukin-1β (IL-1β), and interleukin-18 (IL-18) are extensively studied. TNF-α has a significant impact on glomerular endothelial permeability and podocyte apoptosis (34), while IL-6 promotes mesangial expansion and glomerular fibrosis through JAK/STAT3 activation (35, 36).

**Figure 4:**
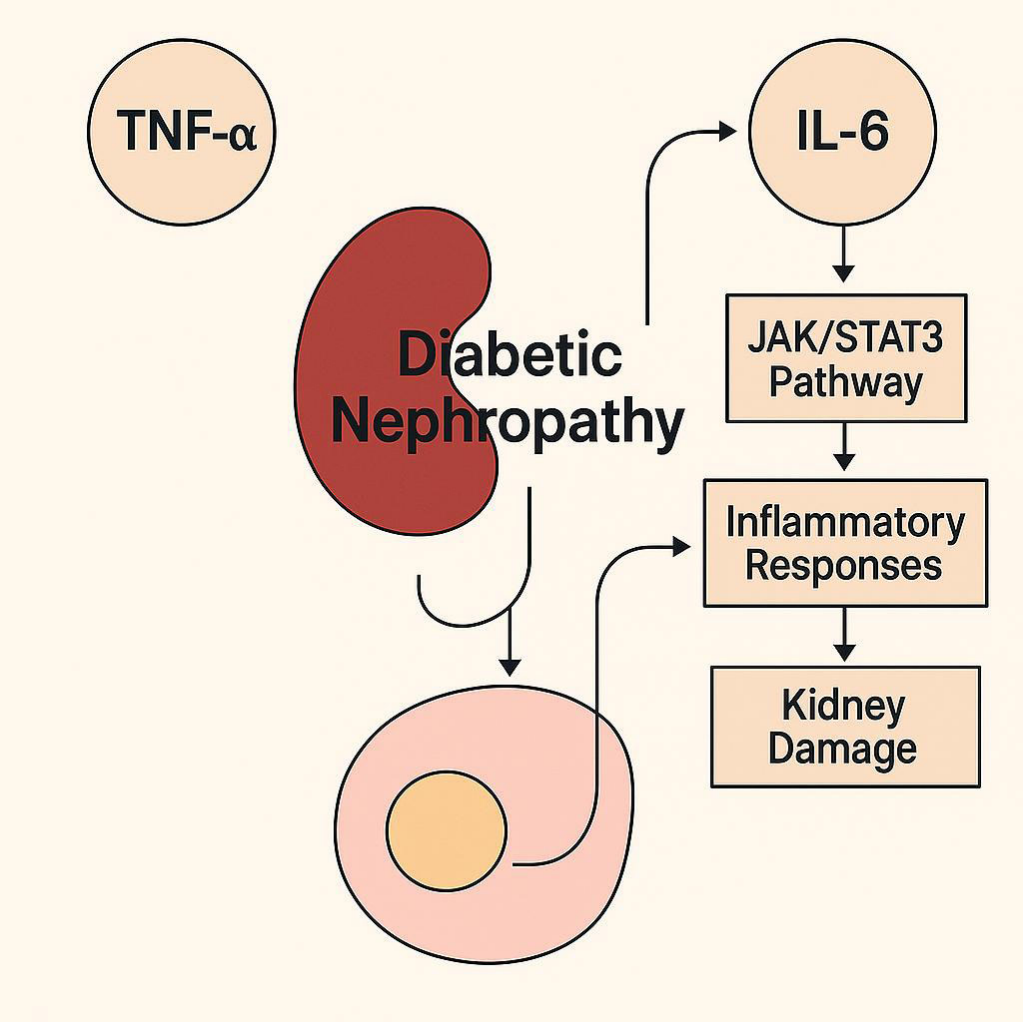
Schematic of cytokine-mediated inflammatory signaling in diabetic nephropathy. TNF-α and IL-6 activate the JAK/STAT3 signaling pathway, leading to enhanced inflammatory responses and subsequent kidney damage. This pathway is a key contributor to the progression of diabetic kidney disease.

IL-18 levels are strongly correlated with tubular injury and are increasingly considered a biomarker of DN severity (37). Additionally, MCP-1 mediates monocyte recruitment into the kidney, perpetuating a local inflammatory environment (4). These cytokines activate downstream inflammatory cascades such as NF-κB and NLRP3 inflammasome, enhancing oxidative stress and apoptosis in renal tissue (5, 38). Clinical data support that elevated serum levels of TNF-α and IL-6 are associated with microalbuminuria and faster progression to ESRD (39). Targeting these cytokines, either directly or via upstream metabolic regulation, represents a viable approach for mitigating DN progression. The role of pro-inflammatory cytokines in diabetic nephropathy is depicted in **Figure 4**.

### 3.4 The relationship between autoimmune response and diabetic nephropathy

**Figure 5:**
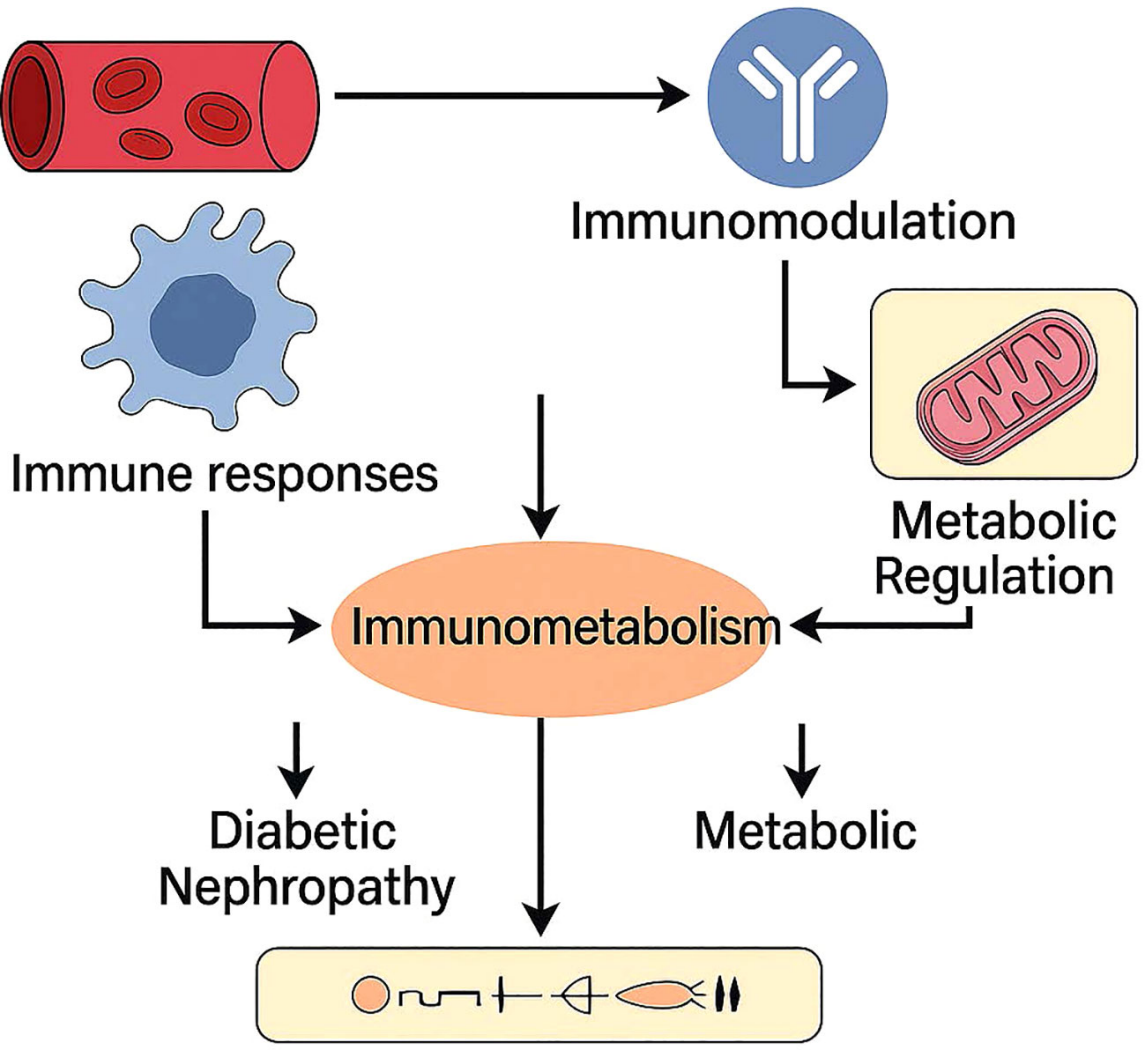
Immunometabolism as a central hub connecting immune responses and metabolic regulation in diabetic nephropathy. Vascular and immune stimuli trigger immunomodulatory and metabolic pathways, which interact through immunometabolic signaling. This crosstalk contributes to the pathogenesis and progression of diabetic nephropathy by exacerbating both immune dysfunction and metabolic stress.

Recent studies have demonstrated the role of the immune system in the onset and progression of diabetic nephropathy(show in **Figure 5**). Research indicates T cell and B cell infiltration within the glomeruli of diabetic nephropathy patients, with glomerular damage closely correlated with this infiltration. The detection of autoantibodies against glomerular components in the serum of diabetic nephropathy patients further suggests these antibodies participate in the kidney damage mechanisms (40).

The immune system in diabetic nephropathy plays roles beyond just cellular immune responses. Patients with this condition show abnormal expression of immunoglobulins, and complement activation through inflammatory responses exacerbates kidney damage (28). Inflammatory factors, particularly IL-18, strongly correlate with the progression of diabetic nephropathy. The expression levels of IL-18 in renal tissue directly correlate with disease progression, highlighting its significance (37). Humoral immune responses also participate throughout the damaging process.

Modifications in immune metabolism are deeply connected to the immune mechanisms involved in diabetic nephropathy, extending beyond basic inflammatory responses. Diabetic patients with hyperglycemia often experience systemic mild inflammation, accompanied by lipid metabolism disorders. Abnormal blood glucose levels, along with impaired lipid metabolism, trigger mild inflammation that affects the entire system in diabetes patients. This long-term inflammatory condition, known as “metaflammation,” has its metabolic basis in the disorders associated with diabetes. Chronic hyperglycemia stimulates inflammatory response pathways, resulting in multiple metabolic and cardiovascular complications (41).

Hyperglycemia activates leukocytes through several pathways, with increased glucose levels leading to the activation of these immune cells. This process contributes to vascular complications in type 2 diabetes mellitus (T2DM). Leukocyte activation markers correlate with elevated glucose levels and insulin resistance, and pro-inflammatory cytokine secretion from hyperglycemia further exacerbates inflammation. This low-grade inflammation persists and characterizes the ongoing condition in diabetic patients.

Lipid metabolic disorders significantly promote inflammation in diabetes. Dysregulation of lipid metabolism, particularly in obesity, attracts immune cells to adipose tissue, which undergoes expansion. Macrophages among these immune cells release inflammatory cytokines, contributing to systemic inflammation associated with metabolic disorders like diabetes (42). The relationship between lipid metabolism and immune responses involves multiple complex mechanisms, including the Wnt signaling pathway and the Toll-like receptor (TLR) pathway, which affect inflammation levels and insulin resistance (43).

This inflammatory state not only accelerates the development of metabolic complications but may also promote the progression of diabetic nephropathy through immune metabolic pathways (25). Therefore, understanding the immune system’s role in diabetic nephropathy is crucial for developing new therapeutic strategies to slow or halt its progression. These immune abnormalities are tightly linked to metabolic dysfunction, reinforcing the concept of immunometabolic synergy in the development of diabetic nephropathy (25).

## 4 Metabolic signaling pathways and diabetic nephropathy

### 4.1 Insulin signaling pathway and diabetic nephropathy

Insulin signaling is crucial for glucose homeostasis, and its disruption can lead to insulin resistance, a key factor in the development of diabetic nephropathy (DN) (**Table 2**). Insulin resistance in the kidney is linked to various metabolic disturbances that contribute to renal injury. In insulin-resistant states, the normal action of insulin on glucose uptake and metabolism is impaired, leading to hyperglycemia and subsequent renal damage. Additionally, the kidney’s role in sodium transport and blood pressure regulation is influenced by insulin signaling pathways, further complicating its involvement in insulin resistance (44).

**Table 2:** Diabetic nephropathy signaling pathways.

Signaling Pathway	Role in Diabetic Nephropathy	Key Mechanisms
Insulin Signaling Pathway	Insulin resistance in the kidney contributes to metabolic dysregulation and kidney damage.	Insulin resistance → impaired glucose uptake → oxidative stress → kidney damage.
PI3K/Akt, mTOR Pathways	Regulates cell growth, survival, and metabolism; dysregulation is linked to kidney fibrosis and inflammation.	mTOR activation → increased protein synthesis → podocyte injury → DN progression.
Wnt/β-catenin Pathway	Involved in kidney fibrosis and epithelial-to-mesenchymal transition (EMT), promoting renal dysfunction.	β-catenin accumulation → fibrosis → mesenchymal transition → renal injury.
JAK/STAT Pathway	Mediates inflammatory responses and immune cell activation in DN progression.	STAT activation → cytokine production → immune cell recruitment → inflammation.
NF-κB Pathway	Key regulator of inflammation and oxidative stress, contributing to kidney damage.	NF-κB activation → production of pro-inflammatory cytokines → chronic kidney inflammation.
Metabolites’ Impact on Immune-Inflammatory Response	Metabolic byproducts like fatty acids and AGEs activate inflammatory pathways, exacerbating DN.	Lipid accumulation & AGEs → oxidative stress → activation of inflammatory cytokines.

During the development of diabetic nephropathy, significant damage occurs as insulin signaling function decreases in the kidney, particularly affecting podocytes. Podocytes are specialized cells in the kidney that maintain and regulate the filtration barrier. When insulin signaling is defective, podocytes begin to malfunction, and their number decreases, a characteristic sign of diabetic nephropathy. This process produces symptoms resembling human diabetic nephropathy when insulin receptors on podocytes are removed, highlighting the significance of insulin signaling in these cells (45).

Insulin resistance, a feature of chronic kidney disease (CKD), disrupts the insulin signaling cascade. This dysfunction involves multiple interconnected signaling pathways that contribute to CKD in an insulin-resistant state. Reduced phosphorylation of key signaling molecules, such as Akt, leads to metabolic abnormalities in glucose, protein, and lipid metabolism, accelerating kidney disease progression (46).

Inflammation and oxidative stress are significant contributors to insulin resistance and diabetic nephropathy. Active inflammatory pathways promote the generation of reactive oxygen species (ROS), which worsen insulin resistance and cause renal tissue damage. Research on the nucleotide-binding oligomerization domain containing 2 (NOD2) suggests its central role in linking inflammation and podocyte insulin resistance in diabetic nephropathy. NOD2 activation reduces insulin signaling effectiveness in podocytes, impairing glucose uptake and leading to renal tissue damage (27).

Genetic factors, such as polymorphisms in genes related to insulin signaling, can also influence the risk of developing diabetic nephropathy. For example, the peroxisome proliferator-activated receptor γ (PPARγ) Pro12Ala polymorphism has been associated with a decreased risk of diabetic nephropathy, suggesting a protective role in insulin resistance and renal function (47).

**Figure 6:**
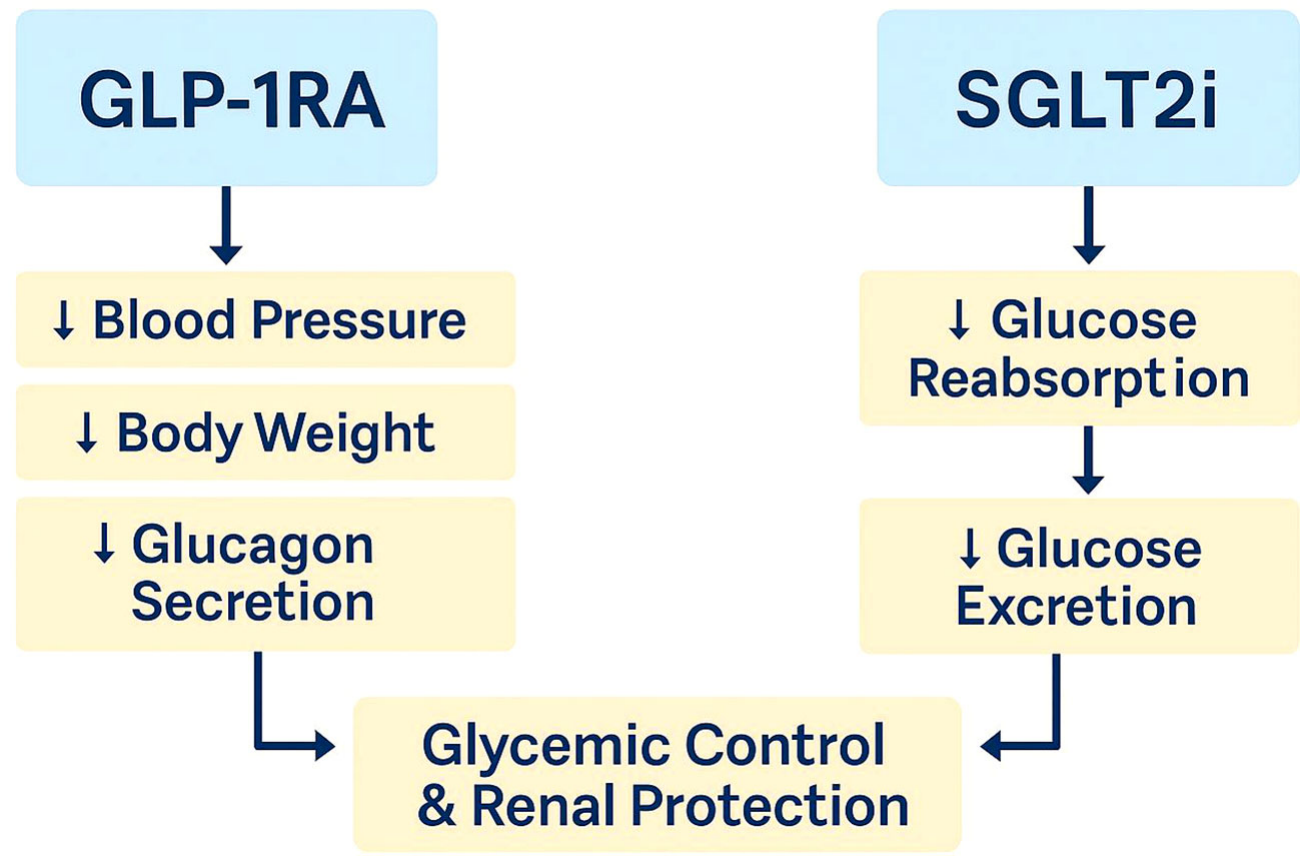
Mechanisms of renoprotective effects of GLP-1 receptor agonists (GLP-1RA) and sodium-glucose cotransporter 2 inhibitors (SGLT2i). GLP-1RA lowers blood pressure, body weight, and glucagon secretion, while SGLT2i reduces glucose reabsorption and enhances glucose excretion. Both contribute to improved glycemic control and renal protection in patients with diabetic nephropathy.

Medical interventions targeting insulin resistance and related pathways have shown beneficial effects in diabetic nephropathy treatment. These interventions aim to enhance insulin signaling in the kidney and reduce inflammation, thereby slowing the progression of renal disease in diabetic patients (48). Understanding the connections between insulin signaling, metabolic disturbances, and renal injury is crucial for developing successful treatments for diabetic nephropathy. Understanding insulin signaling from a cell-type and pathway-specific perspective enables the design of more effective interventions. Future approaches may benefit from precision-guided delivery systems targeting insulin-resistant podocytes and tubular cells in a cell-selective manner(show in **Figure 6**).

Summary of key intracellular signaling pathways implicated in the pathogenesis of diabetic nephropathy. Each pathway contributes to renal injury through distinct but overlapping mechanisms, including oxidative stress, inflammation, fibrosis, and immune activation. Highlighted pathways include PI3K/Akt/mTOR, Wnt/β-catenin, JAK/STAT, and NF-κB, along with the modulatory effects of metabolic byproducts on immune-inflammatory responses.

### 4.2 PI3K/Akt, mTOR and other signaling pathways in relation to diabetic nephropathy

The PI3K/Akt/mTOR signaling cascade is central to regulating cellular processes such as growth, metabolism, autophagy, and survival. In diabetic nephropathy (DN), aberrant activation of this pathway contributes to disease progression by promoting oxidative stress, inflammation, and renal fibrosis. Autophagy, a protective mechanism for removing damaged cellular components, is critically dependent on proper mTOR regulation. Impaired autophagic flux in DN exacerbates mitochondrial dysfunction and oxidative damage, further aggravating insulin resistance and renal injury. Pharmacological inhibition of mTOR has been shown to restore autophagy and ameliorate kidney damage in experimental models (49, 50).

Multiple therapeutic agents exert renoprotective effects via modulation of the PI3K/Akt/mTOR axis. For example, quercetin alleviates apoptosis in tubular epithelial cells by regulating this pathway (51), while SGLT2 inhibitors reduce mTORC1 activity in proximal tubular cells, yielding both glycemic and non-glycemic benefits including anti-fibrotic effects (52). In the realm of traditional medicine, Radix astragali fermented with Paecilomyces cicadae activates podocyte autophagy and confers renal protection (53), and butyrate improves metabolic and muscular outcomes in DN by modulating the pathway through FFA2 signaling (54). Ginkgo biloba extract further demonstrates anti-fibrotic efficacy by suppressing Akt/mTOR signaling (55).

**Figure 7:**
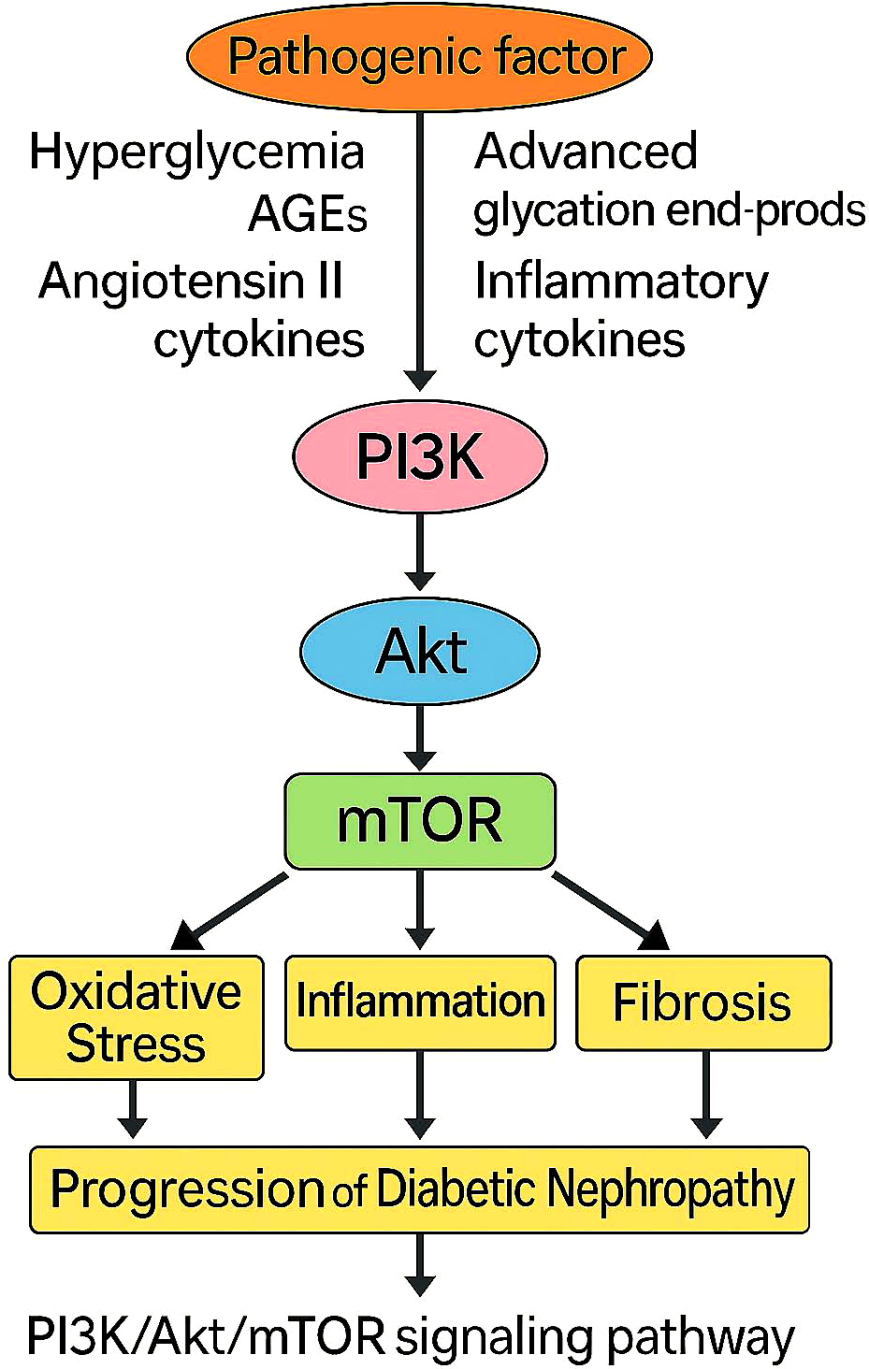
The PI3K/Akt/mTOR signaling pathway involved in the development of diabetic nephropathy. Pathogenic stimuli such as hyperglycemia, AGEs, angiotensin II, and inflammatory cytokines activate the PI3K/Akt/mTOR axis, leading to oxidative stress, inflammation, and fibrosis. These downstream effects collectively contribute to the progression of diabetic nephropathy.

Importantly, the PI3K/Akt/mTOR axis does not act in isolation. It engages in dynamic crosstalk with inflammation-related pathways such as NF-κB and AMPK, creating a metabolic-inflammatory feedback loop that governs epithelial injury and immune activation. This interaction forms the basis of immunometabolism, a concept that integrates immune responses with metabolic regulation in disease pathogenesis. As illustrated in **Figure 7**, immune modulation, mitochondrial dynamics, and systemic metabolic dysfunction converge via immunometabolic signaling, ultimately amplifying renal inflammation and structural deterioration in DN.

Collectively, these findings underscore the dual role of PI3K/Akt/mTOR in mediating both metabolic and immune responses. Targeting this integrative axis holds promise not only for halting renal damage but also for enhancing systemic metabolic balance. Natural compounds such as quercetin and Ginkgo biloba inhibit mTOR-mediated apoptosis and fibrosis (51, 55), while agents like butyrate and Radix astragali provide additional benefits by restoring immunometabolic homeostasis through autophagy and gut–kidney axis modulation (53, 54).

### 4.3 Role of Wnt/β-catenin pathway, JAK/STAT pathway, and NF-κB pathway in diabetic nephropathy

The Wnt/β-catenin signaling pathway plays a critical role in the pathogenesis of diabetic nephropathy. Studies have shown that this pathway is activated in the kidneys of diabetic models, leading to increased inflammation and fibrosis. Suppressing the Wnt signaling pathway can reduce kidney inflammation, fibrosis, and proteinuria (56). Dysregulated activation of the Wnt/β-catenin signaling pathway can also result in pathological changes in renal tubular epithelial cells, exacerbating the progression of diabetic nephropathy (57).

The JAK/STAT signaling pathway also plays a crucial role in diabetic nephropathy. This pathway regulates multiple growth factor and cytokine receptors and modulates inflammatory responses. Overactivation of the JAK/STAT pathway in diabetic nephropathy can cause renal tissue damage and dysfunction. Inhibiting this pathway can help reduce inflammation-induced tissue damage and prevent functional decline (58).

The NF-κB signaling pathway contributes to diabetic nephropathy primarily through its role in inflammation. Activation of NF-κB stimulates the secretion of inflammatory cytokines, exacerbating renal inflammation. Research has also revealed a complex interplay between the NF-κB and Wnt/β-catenin pathways, which may play a significant role in the inflammatory mechanisms underlying diabetic nephropathy (59).

In conclusion, the Wnt/β-catenin, JAK/STAT, and NF-κB signaling pathways interact in the pathogenesis of diabetic nephropathy, collectively influencing disease progression. Investigating the interactions and mechanisms of these pathways may lead to the identification of new therapeutic targets and strategies for treating diabetic nephropathy. Integrated modulation of these intersecting pathways could yield synergistic benefits. For instance, dual inhibition of STAT3 and NF-κB has shown additive anti-fibrotic effects in preclinical DN models, suggesting a viable multi-target strategy for future drug development.

### 4.4 Impact of metabolites on the immune-inflammatory response

Recent studies have focused on the role of metabolites in determining immune-inflammatory reactions. Short-chain fatty acids (SCFAs) play a primary role in immune system regulation by influencing gene expression while also serving as an energy source for colonic and ileal cells. SCFAs strengthen intestinal epithelial barrier mechanisms and affect various innate immune cells, including macrophages, neutrophils, and dendritic cells, all of which contribute to immune system regulation (60). In addition to energy production, fatty acids function as signaling molecules that regulate both metabolism and physiological functions. These molecules are essential for synthesizing complex lipids and act as precursors that influence inflammation. SCFAs also impact insulin sensitivity (61).

The metabolism of fatty acids is tightly regulated, and irregularities in this process can lead to metabolic disorders. Research has shown that medium-chain fatty acids (MCFAs) regulate both glucose and lipid metabolism, opening potential therapeutic avenues for treating metabolic and neurological disorders. Together with glucose metabolites, fatty acids play a fundamental role in this process.

Sugar metabolites are also necessary for regulating immune responses. Research highlights the importance of metabolic intermediates such as lactic acid, acetyl-CoA, and succinate in immune signaling. These metabolites exhibit varying functions depending on their concentrations inside and outside of cells, as well as their location in different subcellular compartments. Metabolites help detect conditions in the microenvironment, enabling cellular adjustments to stress responses and facilitating communication between cells, which contributes to self-regulation (62).

Regulation of immune functions also requires metabolites generated by the gut microbiota. Through metabolic and synthetic pathways, gut microbes modify lipids into bioactive signaling molecules, affecting the host’s physiology. These lipid metabolites alter immune and metabolic processes in the body (63). Research has shown multiple types of immune-inflammatory interactions between metabolites, providing a foundation for developing new therapeutic methods. Decoding the metabolite-immune interface may reveal novel biomarkers and therapeutic entry points for DN, especially in the context of microbiome-targeted and diet-based interventions.

## 5 Interaction of the immune-inflammatory response with metabolic signaling pathways

### 5.1 Mechanisms of interaction between inflammation and metabolic signaling pathways

The interaction between inflammatory and metabolic signaling pathways is a major focus in biomedical research. Emerging evidence highlights the nuclear factor κB (NF-κB) signaling pathway as a key player in both immune modulation and the orchestration of metabolic shifts linked to inflammation and immune responses(**Table 3**). NF-κB regulates a cascade of signaling reactions and collaborates with NF-κB-mediated transcription events to control various metabolic processes. It primarily targets metabolic hormones such as insulin and glucagon, the cellular energy sensor 5’ AMP-activated protein kinase (AMPK), mTOR, as well as numerous metabolic enzymes and regulatory factors. Conversely, metabolic enzymes and their byproducts exert multi-tiered regulation over NF-κB activity, creating a tightly interwoven regulatory circuit (29).

**Table 3:** Interaction Between Immune Inflammation And Metabolic Pathways in DN.

Interaction Aspect	Key Pathways Involved	Mechanisms	Impact on DN Progression
Crosstalk Between Inflammation and Metabolic Signaling	NF-κB, JAK/STAT, PI3K/Akt, AMPK	Chronic inflammation disrupts insulin signaling, leading to metabolic dysfunction and oxidative stress.	Exacerbates metabolic imbalance and inflammation, leading to renal fibrosis.
Immune-Inflammation and Metabolic Remodeling in DN	mTOR, Wnt/β-catenin, TGF-β, oxidative stress pathways	Inflammatory cytokines alter metabolic homeostasis, promoting lipid accumulation and fibrosis.	Drives structural and functional changes in the kidney, accelerating nephropathy.
Interplay of Inflammatory and Metabolic Factors in DN Progression	AGE-RAGE, TNF-α, IL-6, Insulin resistance pathways	Pro-inflammatory cytokines (TNF-α, IL-6) enhance metabolic stress, leading to progressive kidney damage.	Sustains a vicious cycle of inflammation and metabolic stress, worsening DN severity.

Advances in immunometabolism have revealed key interactions between metabolic pathways and immune cell functions (64) (65). Macrophages and T cells undergo dynamic shifts in their metabolic pathways during activation and differentiation, significantly influencing the progression of plaques. Metabolic intermediates play a crucial role in regulating immune cell behavior, contributing to the development of atherosclerosis. By exploring the metabolic regulation of immune responses in atherosclerosis, a field known as atherosclerotic immunometabolism, researchers aim to uncover innovative strategies for prevention and treatment (66).

In the context of obesity and metabolic syndrome, chronic low-grade systemic inflammation plays a significant role in the pathogenesis of obesity and related diseases. The complex signaling networks involved in metabolic inflammation help balance heat production and energy homeostasis, with certain pathways promoting weight gain while others exert an opposing influence. Therefore, modulating immune responses to metabolic inflammation, rather than focusing solely on anti-inflammatory measures to suppress it, could provide a more effective approach for increasing heat production and treating or preventing obesity and its associated diseases (67).

### 5.2 Immunoinflammation and metabolic remodeling in diabetic nephropathy

Diabetic nephropathy (DN), a common complication in individuals with diabetes, arises from complex pathophysiological processes driven by a combination of immune-mediated inflammation and metabolic alterations. Recent studies have increasingly highlighted the critical role of immune system activation and inflammatory responses in the pathogenesis of DN (68). For example, Toll-like receptors (TLRs) play a key role in modulating immune responses and contributing to inflammatory conditions. In diabetic nephropathy, the increased expression of TLR4 is closely associated with tubular inflammation (69). Additionally, NOD2, a key component of the NOD-like receptor family, significantly contributes to the pathological process of diabetic nephropathy; its deficiency can reduce renal damage in diabetic mice (27).

In terms of metabolic remodeling, the progression of diabetic nephropathy is strongly linked to metabolic dysfunction (70) (71). Studies have shown that disorders in glucose and lipid metabolism, coupled with chronic inflammation, are the primary pathological mechanisms driving renal fibrosis in diabetic nephropathy (72). Furthermore, the anti-inflammatory role of microRNAs, such as miR-146a, is gradually being revealed in diabetic nephropathy. Their upregulation can inhibit inflammasome gene activation by downregulating inflammatory-related genes, thereby exerting a protective effect in the early stages of diabetic nephropathy (38).

The occurrence and development of diabetic nephropathy are strongly influenced by the interplay between immune inflammation and metabolic remodeling. Advancements in understanding these mechanisms are increasing scientific knowledge about the pathological processes of DN, suggesting potential new treatment strategies. Alternative approaches to treating diabetic nephropathy may focus on targeting metabolic pathway alterations and regulating immune responses.

### 5.3 Inflammatory and metabolic interplay in the regulation of diabetic nephropathy progression

Inflammatory and metabolic factors play pivotal roles in the progression of diabetic nephropathy. Research has shown that inflammatory responses are key drivers in the development and progression of the disease. Inflammatory factors such as interleukins (IL-1, IL-6, IL-18) and tumor necrosis factor (TNF-α) are activated in diabetic nephropathy, promoting kidney damage through various signaling pathways (73) (74). Additionally, metabolic factors such as hyperglycemia and lipid metabolism disorders significantly influence the pathological process of diabetic nephropathy (75) (76).

Elevated blood glucose levels induce cellular metabolic dysregulation through the activation of the polyol pathway, protein kinase C (PKC) signaling, and the generation of advanced glycation end products (AGEs), all of which accelerate kidney damage (5). Lipid metabolism disorders exacerbate renal tubular interstitial injury by promoting inflammatory responses and oxidative stress (77).

Furthermore, microRNAs, such as miR-146a, play a crucial role in regulating the inflammatory response in diabetic nephropathy. miR-146a exerts a protective effect in the early stages of diabetic nephropathy by downregulating the expression of inflammation-related genes and inhibiting the activation of inflammasome genes (38). Additionally, circular RNAs (circRNAs) are believed to regulate gene expression in diabetic nephropathy, influencing both transcriptional and post-transcriptional levels, thus modulating inflammatory and metabolic processes (73). In sum, the inflammatory-metabolic axis acts as a self-sustaining amplifier of renal injury in DN. Therapeutic strategies aimed at decoupling this axis—such as targeting inflammasome sensors or restoring mitochondrial function—may provide disease-modifying benefits.

## 6 Clinical management and treatment of diabetic nephropathy

### 6.1 Use of hypoglycemic and anti-inflammatory drugs

In recent years, various new types of antidiabetic medications have been developed to help patients control their blood glucose levels through different mechanisms. The administration of glucose-lowering agents and anti-inflammatory medications is central to current therapeutic approaches for diabetes management. Antidiabetic drugs based on incretins, such as GLP-1 receptor agonists and DPP-4 inhibitors, have been shown to demonstrate anti-inflammatory effects (78). These medications reduce blood sugar levels while potentially decreasing the risk of long-term diabetic complications.

Traditional antidiabetic drugs, such as Metformin and sulfonylureas, continue to serve as foundational treatments. However, research into diabetes pathophysiology has led to the emergence of new treatment alternatives. Studies on nanotechnology have shown significant results, particularly with artificial pancreas systems and islet cell transplantation. These medical methods improve diabetes control by increasing drug bioavailability and decreasing toxicity (79).

Chronic inflammation is a principal factor in the development and progression of type 2 diabetes, and anti-inflammatory therapies have shown promising results in reducing this inflammation (80) (81). These therapies help improve insulin resistance, with additional beneficial effects on cardiovascular, renal, and ophthalmic complications.

Research on anti-inflammatory drugs for diabetes treatment has gained significant attention (82). Non-steroidal anti-inflammatory drugs (NSAIDs) provide benefits for diabetic patients, and various immunomodulatory treatments also show promise. However, further investigation is needed to explore the functional mechanisms and extended safety profiles of these treatments (83).

### 6.2 Potential therapeutic strategies for targeting immune-inflammatory responses and metabolic signaling pathways

Recent studies have focused on the intersection of immunoinflammatory responses and metabolic signaling pathways. Immune cells undergo metabolic reprogramming during activation and differentiation, which alters their activity and creates potential therapeutic effects. This process, known as immune metabolism, is regulated by multiple signaling pathways that also influence physiological homeostasis (84).

In obesity and related metabolic diseases, immune cell responses in adipose tissue play a crucial role in maintaining metabolic homeostasis (85). Under healthy conditions, immune cells in white adipose tissue primarily protect the body from weight gain and insulin resistance by secreting type 2 cytokines. However, in obesity, these protective immune pathways become dysregulated, leading to harmful type 1 inflammatory responses that impair glucose metabolism (86).

Increasing scientific attention is being directed towards the study of metabolic pathways in cancer immunotherapy. Research has shown that metabolic regulation within the tumor microenvironment can enhance anti-tumor immune responses. Combining metabolic modulators with immunotherapy offers a potential strategy to overcome resistance to immunotherapy. Although this combined approach holds significant therapeutic promise, challenges remain, such as the impact of metabolic modulators on immune cell metabolism and the emergence of resistance mechanisms (87).

Strategies that focus on immune-inflammatory responses and metabolic signaling pathways have demonstrated positive results in treating various diseases. Research into these pathways has led to new treatments that improve functional processes and enhance patient health, thereby improving their quality of life.

### 6.3 Clinical perspectives of emerging drugs in diabetic nephropathy

Recent advances highlight the significant therapeutic potential of GLP-1 receptor agonists (GLP-1RAs) and SGLT2 inhibitors (SGLT2is) in diabetic nephropathy (DN), not only through glycemic control but also via direct renal and immunological effects (88, 89).

GLP-1RAs enhance insulin secretion and reduce glucagon levels, but emerging evidence reveals that they also exert anti-inflammatory effects in diabetic kidneys. Studies have shown that GLP-1RAs inhibit activation of the NLRP3 inflammasome, reduce IL-1β and TNF-α expression, and suppress macrophage infiltration [Kidney Int Rep, 2024]. These actions alleviate renal inflammation and slow fibrosis independently of their metabolic benefits (90, 91).

SGLT2 inhibitors reduce proximal tubular glucose reabsorption and promote glycosuria, but recent research shows that they also preserve renal oxygenation and mitochondrial function. Moreover, SGLT2is downregulate pro-inflammatory cytokines, reduce oxidative stress, and modulate immune cell recruitment, especially in the renal interstitium [Kidney Int Rep, 2024]. They also attenuate tubular hypertrophy and glomerular hyperfiltration, offering protection against progression to ESRD (92, 93).

These immunometabolic effects complement the cardioprotective actions observed in large trials. Clinicians now consider GLP-1RAs and SGLT2is as cornerstone agents for cardiorenal-metabolic syndrome management (92, 94).

### 6.4 Prospects for dual-targeted immune and metabolic therapies

Dual-target therapy, which combines immunomodulation and metabolic regulation, offers significant therapeutic potential for treating diabetic nephropathy. Recent research has explored the impact of metabolic changes on immune system function during the progression of diabetic nephropathy (95). Diabetic nephropathy, a chronic kidney condition caused by diabetes, involves the disruption of multiple metabolic and immune pathways, which contributes to the pathogenesis of the disease.

Current strategies for treating diabetic nephropathy primarily focus on managing blood glucose levels and hypertension. However, recent advances in disease mechanism research have led to the development of new strategies that modulate immune and metabolic pathways to improve treatment outcomes. Metabolic adaptations that affect immune cell function in the kidneys play a crucial role in disease progression (96). Metabolic reprogramming in immune cells serves as a key factor in controlling immune responses (97).

Studies on metabolic pathway modulation to enhance anti-tumor immune responses have made considerable progress in regulating both immune and metabolic pathways. Modifying metabolic processes within the tumor microenvironment improves the function and success rate of immune cells, thereby enhancing immunotherapy results (98). These methods are also applicable to diabetic nephropathy treatment, where regulating metabolic pathways improves immune responses and slows disease progression (99).

Nanotechnology has created new possibilities for dual-targeted therapy in metabolism and immunity. Nanomaterials enable precise delivery of therapies that regulate specific metabolic pathways, improving therapeutic outcomes (100). This approach has shown promising results in diabetic nephropathy treatment, where simultaneous regulation of metabolic and immune pathways provides better control of disease progression (99).

The development of new methods for treating diabetic nephropathy involves combining immune-targeted and metabolic-targeted treatments. Research into the connections between immune and metabolic processes is helping scientists create more accurate and effective treatments, leading to improved patient prognosis. Further studies on the detailed mechanisms of these pathways during disease development will provide better direction for clinical treatments.

## 7 Conclusion

Diabetic nephropathy (DN) has become a leading cause of chronic kidney disease worldwide. Its pathogenesis is multifaceted, involving both pathological and physiological mechanisms. Recent studies have highlighted the critical role of inflammatory responses in the development of DN, demonstrating their significant contribution to its pathological mechanisms. When key metabolic pathways are disrupted, immune-inflammatory activation occurs, exacerbating the progression of diabetic nephropathy (5).

Chronic activation of the immune system characterizes diabetic nephropathy, leading to inflammation and subsequent renal injury. Research has shown that immune cell infiltration in the kidneys of diabetic nephropathy patients, particularly macrophages, strongly correlates with the advancement of the disease (3). Increased circulating levels of pro-inflammatory cytokines further contribute to the progression of DN. Activation of inflammatory signaling pathways in the kidneys results in more kidney damage due to the action of these cytokines (28).

Abnormal changes in metabolic pathways also play a significant role in the pathological mechanisms of diabetic kidney disease. Hyperglycemia and lipid metabolism disorders trigger low-grade systemic inflammation, which promotes metabolic complications and accelerates the progression of diabetic nephropathy (25). Metabolic functions inside cells interact with immune activation, influencing the pathological process of diabetic nephropathy. These interactions between metabolism and immunity regulate immune cell metabolism, impacting disease development.

There is a mutual influence between immune-inflammatory mechanisms and metabolic pathways, which are critical to the pathogenesis and progression of diabetic kidney disease. Research into these mechanisms has improved our understanding of the pathophysiological processes involved in diabetic nephropathy, laying the foundation for the development of new therapeutic approaches. Treatment strategies targeting both inflammatory and metabolic signaling pathways are emerging as potential methods for managing and preventing diabetic nephropathy.

Future research should focus on the mechanisms of immune-metabolic interactions, as immune cell function is strongly influenced by their metabolic activities. Modifying metabolic processes holds promise for treating immune-related disorders, with studies showing that targeting metabolic pathways can eliminate cancer cells and modulate immune responses in autoimmune diseases. Additionally, regulating metabolic homeostasis is essential for the proper function of innate immune cells, especially in obesity-related type 2 diabetes. The gut microbiome’s composition and its metabolic activities offer opportunities for personalized treatment strategies. While implementing precision medicine based on individual metabolic profiles presents challenges in clinical settings, it also holds potential for significant medical advancements. Ongoing research into the gut microbiome-host relationship is expected to lead to more targeted treatments. Although the field is still in its early stages, it shows considerable promise for clinical applications. Immunotherapy for blood-related diseases continues to evolve, offering new treatment options despite remaining barriers.

Increased research into immune-metabolic interactions is advancing our understanding of disease pathogenesis and driving the development of alternative treatments. This progress is essential for creating personalized therapeutic strategies, which will be the focus of future studies.
